# Effect of acute alcohol consumption on blunt bowel mesenteric injury: a retrospective analysis

**DOI:** 10.1186/s12873-023-00928-1

**Published:** 2024-01-07

**Authors:** Ting-Min Hsieh, Kuo-Chen Huang, Po-Chun Chuang, Chun-Ting Liu, Bei-Yu Wu, Ching-Hua Hsieh, Fu-Jen Cheng

**Affiliations:** 1 Division of Trauma, Department of Surgery, Kaohsiung Chang Gung Memorial Hospital, Chang Gung University College of Medicine, 123 Ta Pei Road, Niao-Song District, Kaohsiung, Taiwan; 2 Department of Emergency, Kaohsiung Chang Gung Memorial Hospital, Chang Gung University College of Medicine, 123 Ta Pei Road, Niao-Song District, Kaohsiung, Taiwan; 3 Department of Chinese Medicine, Kaohsiung Chang Gung Memorial Hospital, Chang Gung University College of Medicine, 123 Ta Pei Road, Niao-Song District, Kaohsiung, Taiwan; 4 Division of Plastic Surgery, Department of Surgery, Kaohsiung Chang Gung Memorial Hospital, Chang Gung University College of Medicine, 123 Ta Pei Road, Niao-Song District, Kaohsiung, Taiwan

**Keywords:** Alcohol, Blunt abdomen trauma, Hemodynamic, Emergency department

## Abstract

**Background:**

The effect of alcohol consumption on trauma remains controversial. The effects of alcohol on hemorrhage and peritonitis after blunt abdominal trauma have rarely been discussed. This study aimed to explore the effects of acute alcohol intoxication on the clinical characteristics, injury patterns, and outcomes in a surgical blunt bowel mesenteric injury (BBMI) cohort.

**Methods:**

A retrospective data analysis was performed using trauma cases of patients who had been tested for alcohol and had surgically proven BBMI from a Trauma Registry System from 2009 to 2021. Patients were grouped according to their positive blood alcohol concentration (BAC; >0.5% vs. no BAC; less than 0.5% no BAC) upon arrival at the emergency department (ED). The injury characteristics, physiological parameters, and outcomes with respect to post-injury complications and mortality were assessed.

**Results:**

In total, 142 patients with surgical BBMI were included. Of these, 116 and 26 patients were assigned to the BAC-negative and BAC-positive groups, respectively. The overall injury severity, injury pattern, and age were comparable between the groups. The patients in the BAC-positive group had a significantly lower systolic blood pressure (99 mmHg vs. 119 mmHg; *p* = 0.046), worse shock index (0.96 vs. 0.82; *p* = 0.048), and lower percentage and number of packed red blood cells transfused (34.6% vs. 57.8%; *p* = 0.032 and 0 U vs. 2 U; *p* = 0.031) than those in the BAC-negative group. Additionally, although not statistically significant, patients in the BAC-positive group had lower leukocyte counts (9,700 cells/mm^3^ vs. 11,600 cells/mm^3^; *p* = 0.165 ) at the ED. However, significantly reduced percentages of leukocytes ≥ 12,000 cells/mm^3^ (26.9% vs. 48.3%; *p* = 0.048) and ≥ 12,000 or ≤ 4,000 cells/mm^3^ (26.9% vs. 50.9%; *p* = 0.027) were observed in the BAC-positive group at the ED. Furthermore, the 30-day mortality rate did not show statistically significant differences, and there was a higher incidence of bowel-related mortality in the BAC-positive group (11.5% vs. 1.7%, *p* = 0.043).

**Conclusions:**

For patients with BBMI arriving alive to the hospital, acute alcohol consumption was associated with significantly worse hemodynamic parameters, interfered inflammation status, and higher bowel related mortality rate.

## Introduction

Alcohol consumption is usually thought to have adverse effects on society and often occurs as a result of trauma. Cases of acute alcohol exposure are not uncommon in the emergency department (ED), accounting for 45–53% of adult patients admitted with trauma [1, 2] and contributing to approximately one-third of trauma-related deaths [3]. However, the influence of alcohol on trauma has provided inconsistent results on the injury severity score (ISS), length of hospital stay, morbidity, and mortality [1–7]. Some studies have reported that the condition of patients with trauma with acute alcohol intoxication (AAI) was consistent with the theory of “loose or relax” status with less injury severity and reduced mortality; therefore, these studies have concluded that there is a protective effect and survival benefit amongst patients with trauma and AAI [8–10]. Despite these findings, other studies have postulated that this might be due to a falsely inflated ISS in patients with AAI, suggesting a pseudo-protective effect of alcohol on trauma [11]. Studies have suggested that alcohol leads to more severe head injury [9–11], resulting in a number of studies exploring the relationship between AAI and traumatic brain injury (TBI), which concluded with a theory of neuroprotection in TBI with AAI [7, 8]. In contrast, other studies have not supported the theory of neuroprotective effects of alcohol in TBI [12]. Given the effect of alcohol on the immune system documented in in-vivo or in-vitro tests [13, 14], studies have reported higher infection and morbidity in patients with trauma and AAI and concluded that alcohol has an immunosuppressive effect on trauma [15]. However, other studies [16, 17] considered that although alcohol could lead to immunosuppression in patients with trauma, this was not associated with post-traumatic infective complications, such as sepsis, multiple organ dysfunction, and systemic inflammatory response syndrome (SIRS). Despite the clinical evidence in human studies, Brigodeetl et al. [12] did not support this theory and reported that alcohol-intoxicated patients with TBI had fewer infectious complications, perhaps due to the immunomodulatory effect of alcohol in TBI. Although the association between alcohol consumption and trauma has been extensively studied, and some studies have found a significantly higher incidence of abdominal injuries in patients with polytrauma and AAI [2, 5], the relationship between alcohol consumption and abdominal trauma is rarely discussed.

Abdominal trauma is the third most common anatomical injury, and approximately a quarter of these cases require surgery [18]. In contrast to penetrating abdominal trauma, significant blunt abdominal trauma (BAT) may go undetected and be easily overlooked, especially when the patient is intoxicated with alcohol, which would lead to significant morbidity and mortality.

However, only two retrospective studies by Gentilello et al. [15] and Benson et al. [19] in 1993 and 2018, respectively, have specifically addressed the effect of alcohol on patients with abdominal trauma who had undergone emergency laparotomy. One study in patients with abdomen-penetration trauma with hollow viscus injury demonstrated a depressed post-traumatic immunologic response, leading to a 2.6 fold increased risk of trauma-related infection in these patients [15]. Another study compared alcohol-positive and alcohol-negative patients with trauma requiring emergency laparotomy and found no major difference in complications and mortality, even after controlling for age, sex, ISS, and mechanism of injury between the groups [19]. However, the latter study included approximately half the number of patients with predominant penetration injuries (56%). Given the clinical importance and contradictory results in the existing studies, we sought to test the impact of alcohol on patients with BAT.

Blunt bowel/mesenteric injuries (BBMIs) cover the third most vulnerable organ in BAT after the spleen and liver and are among the few indications for emergency laparotomy in this era of non-operative management [20]. The pathophysiologic effect of BBMI involves not only fatal mesenteric bleeding but also subtle peritonitis from hollow organ injuries. In animal experiments, AAI had an immunosuppressive effect on hemorrhagic shock in a rat model [13, 21], whereas AAI induced the early development of shock and renal dysfunction in peritonitis in an ovine model [22]. Conducting a clinical study on the effect of alcohol on patients is difficult because of the multiplicity and variability of the patient factors. We hypothesized that AAI would acutely impair immunity and increase the risk of hemodynamic instability during the resuscitation of patients with trauma. The concomitant presence of alcohol consumption, internal bleeding, and bacterial contamination peritonitis in patients with BBMI provides an opportunity to test this hypothesis. This study aimed to determine the differences in the clinical presentation, injury patterns, and outcomes of morbidity and mortality between alcohol-positive and alcohol-negative patients with BBMI.

## Methods

### Ethics statement

Approval for this research was granted by the Institutional Review Board (IRB) at Chang Gung Memorial Hospital (approval code: 201902275B0). The necessity for obtaining informed consent was waived in accordance with IRB regulations.

### Study population

**Fig. 1:**
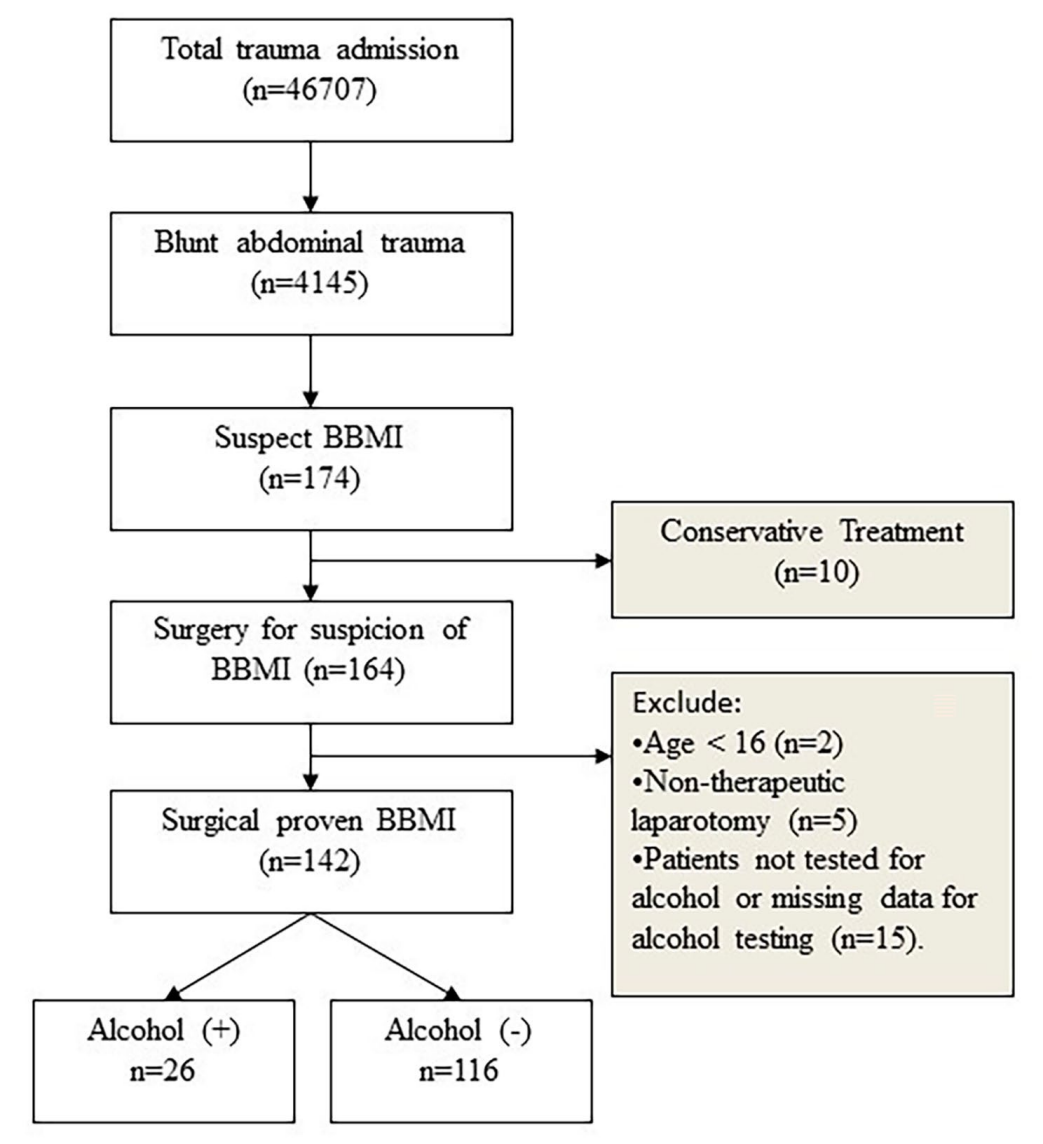
Enrollment process flowchart for patients with surgical blunt mesenteric bowel injury (BBMI) BBMI: blunt bowel mesentery injury

This retrospective investigation encompassed the comprehensive dataset from the Trauma Registry System, spanning the timeframe from January 1, 2009, to December 31, 2021, within a medical facility housing 2,686 beds. The facility serves as a Level I regional trauma center, catering to trauma patients in the southern region of Taiwan [6, 23–25]. All data were sourced from prospective records of hospitalized adult trauma patients and subjected to subsequent retrospective analysis. The scope of the study encompassed patients who had undergone emergency laparotomy for suspected blunt bowel and mesenteric injury (BBMI) and had been screened for alcohol consumption. Immediate measurement of serum alcohol levels was conducted upon admission. Subsequent to diagnostic assessment through surgical exploration, all enrolled patients were confirmed to have incurred either gastrointestinal tract or mesenteric injuries during the procedure. The study also extended to encompass patients with injuries to the stomach, duodenum, or rectum. Exclusion criteria consisted of patients below the age of 16, those lacking documented blood alcohol concentration (BAC) test outcomes or incomplete alcohol consumption data, individuals who expired within the emergency department, those managed conservatively without surgical intervention, and cases involving non-therapeutic laparotomy prompted by suspected BBMI. The therapeutic laparotomy involved procedures such as bowel resection, enterorrhaphy, enterostomy, or mesenteric repair, primarily targeting BBMI. The cohort was categorized based on BAC levels into two groups: the positive-BAC group (BAC > 50 mg/dL) and the negative-BAC group (BAC < 50 mg/dL). The selection procedure for the study sample is visually delineated in Fig. 1.

### Study parameters

Collected patient information and variables as illustrated in previous study [20]. Briefly, demographic data, clinical and trauma data consisted of trauma scores, including ISS, new ISS, trauma resuscitation injury severity score, revised trauma score; vital signs at the ED, injury mechanisms, and clinical presentation, including the laboratory data were recorded from ED database. Additionally, we included the incidence of intubation and tube thoracostomy at the ED; status of shock (defined as SBP of ≤ 90 mmHg); blood transfusion (BT) status before surgical intervention. The operative findings, as documented in the operative records, included an assessment of bowel injuries, which were categorized as follows: isolated bowel injury (involving only small bowel injuries such as ischemia, rupture, serosa injury, or hematoma), isolated colon injury (covering colon injuries, including ischemia, rupture, serosa injury, or hematoma), isolated mesentery injury (encompassing mesenteric injuries, such as ischemia, rupture, serosa injury, or hematoma), and combined injury (involving either small bowel or colon injury concurrent with mesenteric injury, including ischemia, rupture, serosa injury, or hematoma). Furthermore, operative blood loss was evaluated as part of the findings. Outcome metrics encompassed various parameters, including morbidity, mortality, 24-hour mortality rate, mortality associated with bowel-related issues (defined as mortality due to abdomen-related sepsis including bowel ischemic or postoperative anastomosis leakage), mortality due to exsanguination (defined as surgically proven hemorrhagic shock resulting from bowel or mesenteric bleeding), duration of hospitalization, and stay in the intensive care unit (ICU). We also tracked the incidence of abbreviated injury scores (AISs) equal to or exceeding ≥ 2 and ≥ 3 for each anatomical region. The morbidities included post-operative infection, sepsis (assessed using the 2005 criteria outlined by the International Sepsis Forum), and SIRS, ( (to examine the immunosuppressive effect of alcohol in case of peritonitis, we chose the WBC count cut-off value of ≥ 12,000 or ≤ 4,000 cells/mm^3^ to investigate the anti-SIRS effect of alcohol by comparing the difference in the percentages between the two groups) [26], unplanned intubation on admission, complications of operation, such as leakage, wound dehiscence, enterocutaneous fistula, and thromboembolic disorders were also recorded.

### Statistical analysis

The acquired data underwent analysis using IBM SPSS Statistics for Windows (version 20.0; IBM Corp., Armonk, NY). Continuous data were presented as medians along with interquartile ranges, while categorical data were represented as frequencies and corresponding percentages. Comparative analysis of categorical variables for the two groups was conducted using a two-sided Fisher’s exact test or Pearson’s chi-square test. The unpaired Student’s t-test was applied to assess normally distributed continuous variables, and the Mann–Whitney U test was employed for non-normally distributed data. Statistical significance was considered achieved at a threshold of *p* < 0.05.

Kaplan–Meier analysis was utilized to investigate whether the BAC-positive group exhibited a heightened risk of morbidity in comparison to the BAC-negative group. To evaluate distinctions in the morbidity curves between the groups, the log-rank test was employed.

All data analyses were performed using IBM SPSS Statistics for Windows (version 20.0; IBM Corp., Armonk, NY). Continuous data were presented as medians and interquartile ranges, while categorical data were expressed as frequencies and percentages. For categorical variables within the two groups, either a two-sided Fisher’s exact test or Pearson’s chi-square test was used for comparison. The unpaired Student’s t-test was employed for normally distributed continuous variables, and non-normally distributed data were compared using the Mann–Whitney U test. Statistical significance was defined as *p* < 0.05.

To assess whether the BAC-positive group bore a greater morbidity risk than the BAC-negative group, Kaplan–Meier analysis was applied. Differences in morbidity curves between the groups were assessed using the log-rank test.

## Results

### Patient characteristics, clinical presentation, and outcomes

**Fig. 2:**
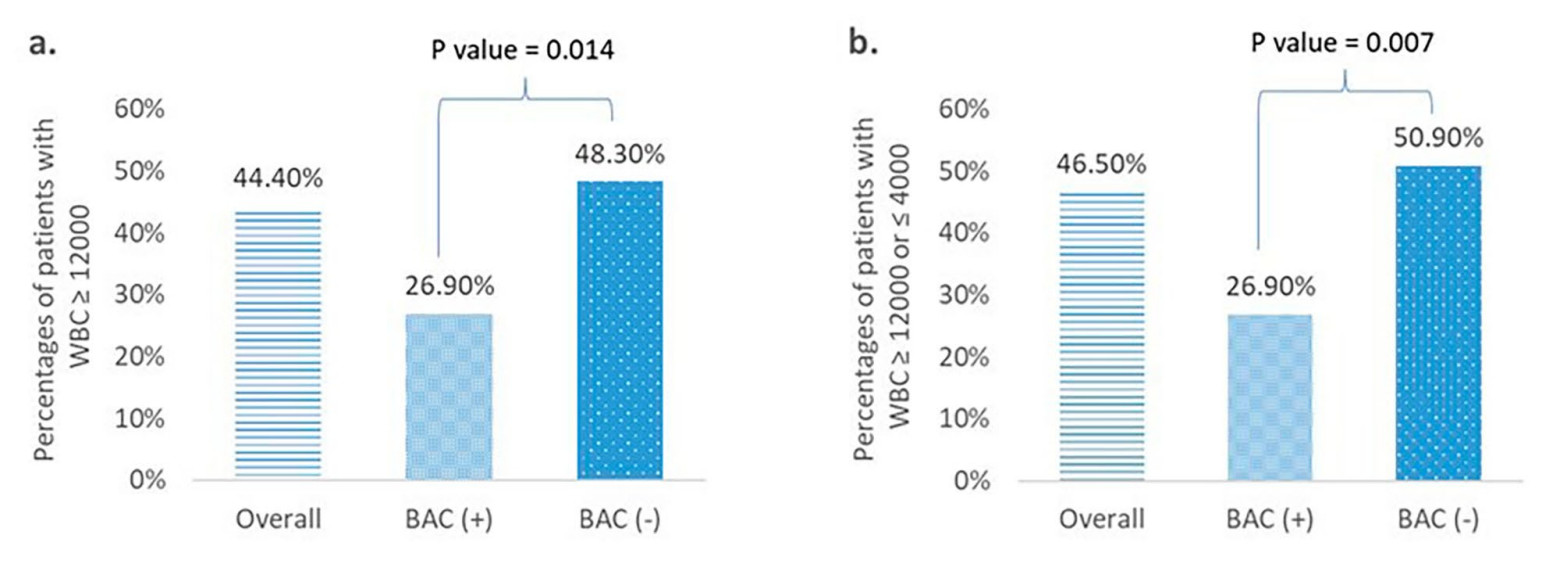
Percentage of patients with **2a**. WBC ≥ 12,000 cells/mm^3^ and **2b**. WBC ≥ 12,000 or ≤ 4,000 cells/mm^3^ on arrival to the emergency department

Overall, 142 patients with trauma admitted to the ED with a surgically proven BBMI were included in this study. Of these, 116 patients were assigned to the BAC-negative group and 26 to the BAC-positive group, with a significant predominance of men than that of women (77.6% vs. 100%, *p* = 0.04). BAC-positive patients had a significantly lower SBP (99 [86–118] mmHg vs. 119 [93–136.5] mmHg, *p* = 0.046) and higher SI (0.96 [0.74–1.44] vs. 0.82 [0.63–1.05], *p* = 0.048) at the ED than the BAC-negative patients. Motorcycle (51.4%) and car (27.5%) accidents were the leading causes of injury for this sample. A greater proportion of those who tested positive for alcohol had had car accidents compared to their alcohol-negative counterparts (50% vs. 22.4%, *p* = 0.045). The average ED WBC count was 10,900 (8,600–16,300) cells/mm^3^. The WBC count in the ED in the BAC-positive group (9,700 [8,000–12,300] cells/mm^3^) was slightly lower than that in the BAC-negative group (11,600 [8,600–16,450] cells/mm^3^); however, this difference was not significant (p = 0.165). To evaluate the influence of SIRS on our results, the proportion of WBCs ≥ 12,000 and ≤ 4,000 cells/mm^3^ was analyzed. The percentage of WBCs ≥ 12,000 cells/mm^3^ was significantly less frequent in BAC-positive patients compared than in BAC-negative patients (26.9% vs. 48.3%, *p* = 0.048), even after adjusting for confounders, such as age, sex, SI, and injury mechanisms (adjusted *p* = 0.014). The percentage of WBCs ≥ 12,000 or ≤ 4,000 cells/mm^3^ was significantly less frequent in BAC-positive patients than in the BAC-negative patients (26.9% vs. 50.9%, *p* = 0.027), even after controlling for confounders, such as age, sex, SI, and injury mechanisms (adjusted *p* = 0.007). (Fig. 2, a and b).

The *P*-value is adjusted by age, sex, shock index, and injury mechanism. WBC, white blood count; BAC, blood alcohol concentration.

Regarding blood transfusion, the BAC-positive group had a significantly lower ratio of packed RBCs transfused in the ED than that for the BAC-negative group (34.6% vs. 57.8%, *p* = 0.032). Additionally, the BAC-positive group received significantly fewer packed RBC units in the ED than those received by the BAC-negative group (0 [0–2] vs. 2 [0–4] U, *p* = 0.031). The differences in the application of packed RBCs within the first 24 h in the operating room or ward and the rates of massive transfusion were comparable. Regarding outcomes, the BAC-positive group had a significantly higher incidence of bowel-related mortality than the BAC-negative group (11.5% vs. 1.7%, *p* = 0.043). Overall morbidity and mortality statistics were not significantly different between the two groups (Table 1).

**Table 1:** Clinical and injury profiles of BBMI patients categorized by alcohol consumption group

	Overall (N = 142)	BAC (+) (n = 26)	BAC (-) (n = 116)	***P*** value
Age	44.5 (30–58)	43 (32–52)	46.5 (28.5–63)	0.289
Male sex	116 (81.7%)	26 (100%)	90 (77.6%)	0.040
ISS (injury severity score)	16 (9–25)	20 (9–24)	16 (9–25)	0.376
ISS ≥ 16	79 (55.6%)	17 (65.4%)	62 (53.4%)	0.268
ISS ≥ 25	36 (25.4%)	6 (23.1%)	30 (25.9%)	0.768
NISS (new ISS)	19 (9–27)	21.5 (9–29)	18 (9–27)	0.688
TRISS (trauma resuscitation ISS)	0.97 (0.934–0.99)	0.98 (0.955–0.988)	0.97 (0.923–0.991)	0.509
RTS (revised trauma score)	7.84 (7.108–7.84)	7.84 (6.904–7.84)	7.84 (7.108–7.84)	0.597
**ED vital sign**				
Temperature	36.3 (36.0-36.9)	36.0 (36.0-36.2)	36.5 (36.1–37.0)	0.001
SBP (mm/Hg)	117.5 (91–134)	99 (86–118)	119 (93-136.5)	0.046
HR (/min)	97.5 (80–117)	101 (87–121)	97 (79.5-115.5)	0.219
HR > 90/min	84 (59.2%)	18 (69.2%)	65 (56.9%)	0.248
RR (/min)	20 (18–20)	20 (19–21)	20 (18–20)	0.122
GCS	15 (15–15)	15 (14–15)	15 (15–15)	0.463
SIRS score	1 (1–2)	2 (1–2)	1 (1–2)	0.936
SIRS ≥ 2	68 (47.9%)	14 (53.8%)	54 (46.6%)	0.501
Shock index (bpm/mmHg)	0.85 (0.65–1.1)	0.96 (0.74–1.44)	0.82 (0.63–1.05)	0.048
**Mechanism**				
Motorcycle (%)	73 (51.4%)	9 (34.6%)	64 (55.2%)	0.045
Car (%)	39 (27.5%)	13 (50%)	26 (22.4%)	
Fall (%)	3 (2.1%)	0 (0%)	3 (2.6%)	
High fall (%)	5 (3.5%)	0 (0%)	5 (4.3%)	
Pedestrian (%)	6 (4.2%)	2 (7.7%)	4 (3.4%)	
Assault (%)	5 (3.5%)	2 (7.7%)	3 (2.6%)	
Bicycle (%)	6 (4.2%)	0 (0%)	6 (5.2%)	
Impact (%)	5 (3.5%)	0 (0%)	5 (4.3%)	
**Clinical presentation**				
ED WBC (cells/mm^3^)	10,900 (8600–16,300)	9700 (8000–12,300)	11,600 (8600–16,450)	0.165
ED WBC ≥ 12,000	63 (44.4%)	7 (26.9%)	56 (48.3%)	0.048
ED WBC ≥ 12,000 or ≤ 4000	66 (46.5%)	7 (26.9%)	59 (50.9%)	0.027
ED hemoglobin (g/dL)	12.6 (10.7–14.2)	13.6 (11.6–15)	12.5 (10.6–13.9)	0.092
ED Intubation (%)	28 (19.7%)	3 (11.5%)	25 (21.6%)	0.246
Chest tube (%)	29 (20.4%)	3 (11.5%)	26 (22.4%)	0.214
Shock before OR (%)	62 (43.7%)	13 (50%)	49 (42.2%)	0.471
**Operative finding**				
Isolated bowel injury (%)	40 (28.2%)	7 (26.9%)	33 (28.4%)	0.876
Isolated colon injury (%)	16 (11.3%)	3 (11.5%)	13 (11.2%)	1.000
Isolated mesentery injury (%)	40 (28.2%)	6 (23.1%)	34 (29.3%)	0.523
Combined injury (%)	47 (33.1%)	11 (42.3%)	36 (31%)	0.270
OP blood loss (ml)	500 (100–2000)	700 (100–2000)	500 (100–1900)	0.729
**Blood transfusion**				
B/T at ED (%)	76 (53.5%)	9 (34.6%)	67 (57.8%)	0.032
ED Pack RBC (U)	2 (0–4)	0 (0–2)	2 (0–4)	0.031
ED FFP (U)	0 (0–2)	0 (0–0)	0 (0-2.5)	0.148
24 h Pack RBC (U)	4 (0–12)	4 (0–12)	4 (0-11.5)	0.629
24 h FFP (U)	2 (0–8)	4 (0–6)	2 (0–8)	0.725
Massive transfusion (%)	43 (30.3%)	8 (30.8%)	35 (30.2%)	0.952
OR Pack RBC (U)	2 (0–6)	1 (0–8)	2 (0–6)	0.942
OR FFP (U)	0 (0–4)	3 (0–4)	0 (0–4)	0.374
Ward pack RBC (U)	0 (0–4)	1 (0–7)	0 (0–2)	0.070
Ward FFP (U)	0 (0–6)	2 (0–11)	0 (0–4)	0.017
**Outcome**				
Morbidity (%)	94 (66.2%)	21 (80.8%)	73 (62.9%)	0.082
Mortality (%)	18 (12.7%)	5 (19.2%)	13 (11.2%)	0.325
24 h mortality (%)	6 (4.2%)	0 (0%)	6 (5.2%)	0.592
Bowel related mortality (%)	5 (3.5%)	3 (11.5%)	2 (1.7%)	0.043
Exsanguination mortality (%)	9 (6.3%)	2 (7.7%)	7 (6%)	0.669
ICU length of stay (day)	3 (2–7)	4 (2–10)	3 (1.5-7)	0.167
Hospitalization LOS (day)	17 (11–32)	22 (15–40)	16 (10–31)	0.141

BBMI: blunt bowel mesentery injury; BAC: blood alcohol concentration; ED: emergency department; SBP: systolic blood pressure; HR: heart rate; RR: respiratory rate; GCS: Glasgow Coma Scale; WBC: white blood cell; B/T: blood transfusion; OR: operative room; ICU: intensive care unit; Data were presented as a number (percentage) and median IQR (25–75%); SIRS: Systemic Inflammatory Response Syndrome

### Injury severity and pattern

The distribution of AIS injuries in each body region in the two groups is shown in Table 2. No significant differences among the groups in terms of AIS scores and incidence of an AIS score of ≥ 2 or ≥ 3 in all body regions were observed.

**Table 2:** Severity of injury in body regions of patients with BBMI according to the alcohol consumption group

	Overall (N = 142)	BAC (+) (n = 26)	BAC (-) (n = 116)	***P*** value
AIS head	0 (0–0)	0 (0–0)	0 (0–0)	0.511
AIS face	0 (0–0)	0 (0–0)	0 (0–0)	0.648
AIS chest	0 (0–1)	0 (0–0)	0 (0–2)	0.541
AIS abdomen	3 (3–4)	3 (3–4)	3 (3–4)	0.799
AIS extremities	0 (0–2)	0 (0–2)	0 (0–2)	0.348
AIS head ≥ 2	19 (13.6%)	5 (19.2%)	14 (12.3%)	0.350
AIS head ≥ 3	14 (10%)	3 (11.5%)	11 (9.6%)	0.724
AIS face ≥ 2	9 (6.4%)	2 (7.7%)	7 (6.1%)	0.673
AIS face ≥ 3	0 (0%)	0 (0%)	0 (0%)	
AIS chest ≥ 2	34 (24.3%)	4 (15.4%)	30 (26.3%)	0.241
AIS chest ≥ 3	32 (22.9%)	4 (15.4%)	28 (24.6%)	0.315
AIS abdomen ≥ 2	140 (100%)	26 (100%)	114 (100%)	
AIS abdomen ≥ 3	129 (92.1%)	24 (92.3%)	105 (92.1%)	1.000
AIS extremities ≥ 2	50 (35.7%)	12 (46.2%)	38 (33.3%)	0.218
AIS extremities ≥ 3	27 (19.3%)	6 (23.1%)	21 (18.4%)	0.587

Data were presented as a number (percentage) and median IQR (25–75%)

AIS: abbreviated injury score; BBMI: blunt bowel mesentery injury; BAC: blood alcohol concentration

### Overall morbidities

Morbidity rates are shown in Table 3.

**Table 3:** Incidence rates of post-injury complications among patients with BBMI according to the alcohol consumption group

	Overall (N = 142)	BAC (+) (n = 26)	BAC (-) (n = 116)	***P*** value
Sepsis	25 (17.6%)	4 (15.4%)	21 (18.1%)	1.000
Pneumonia	22 (15.5%)	6 (23.1%)	16 (13.8%)	0.240
Septic shock	10 (7%)	4 (15.4%)	6 (5.2%)	0.085
Unplanned intubation in ward	14 (9.9%)	6 (23.1%)	8 (6.9%)	0.023
Intraabdominal abscess	16 (11.3%)	4 (15.4%)	12 (10.3%)	0.494
Leakage	9 (6.3%)	2 (7.7%)	7 (6%)	0.669
Coagulopathy	57 (40.1%)	14 (53.8%)	43 (37.1%)	0.115
Acute renal failure	50 (35.2%)	11 (42.3%)	39 (33.6%)	0.402
Acidosis	38 (26.8%)	9 (34.6%)	29 (25%)	0.317
Urinary tract infection	24 (16.9%)	3 (11.5%)	21 (18.1%)	0.568
Stroke	3 (2.1%)	0 (0%)	3 (2.6%)	1.000
Pulmonary embolism	2 (1.4%)	0 (0%)	2 (1.7%)	1.000
ARDS	2 (1.4%)	0 (0%)	2 (1.7%)	1.000
Pleural effusion	23 (16.2%)	3 (11.5%)	20 (17.2%)	0.570
Enterocutaneous fistula	2 (1.4%)	0 (0%)	2 (1.7%)	1.000
Wound infection	32 (22.5%)	9 (34.6%)	23 (19.8%)	0.103
Wound dehiscence	8 (5.6%)	5 (19.2%)	3 (2.6%)	0.005
Abdomen compartment	6 (4.2%)	1 (3.8%)	5 (4.3%)	1.000
Tracheostomy	3 (2.1%)	0 (18.1%)	3 (2.6%)	1.000
ECMO	2 (1.4%)	0 (0%)	2 (1.7%)	1.000
Return to OR	22 (15.5%)	5 (19.2%)	17 (14.7%)	0.555
Hemodialysis	2 (1.4%)	0 (0%)	2 (1.7%)	1.000

BAC: blood alcohol concentration; ARDS: acute respiratory distress syndrome; ECMO: Extracorporeal Membrane Oxygenation; OR: operative room; Data were presented as a number (percentage)

**Fig. 3:**
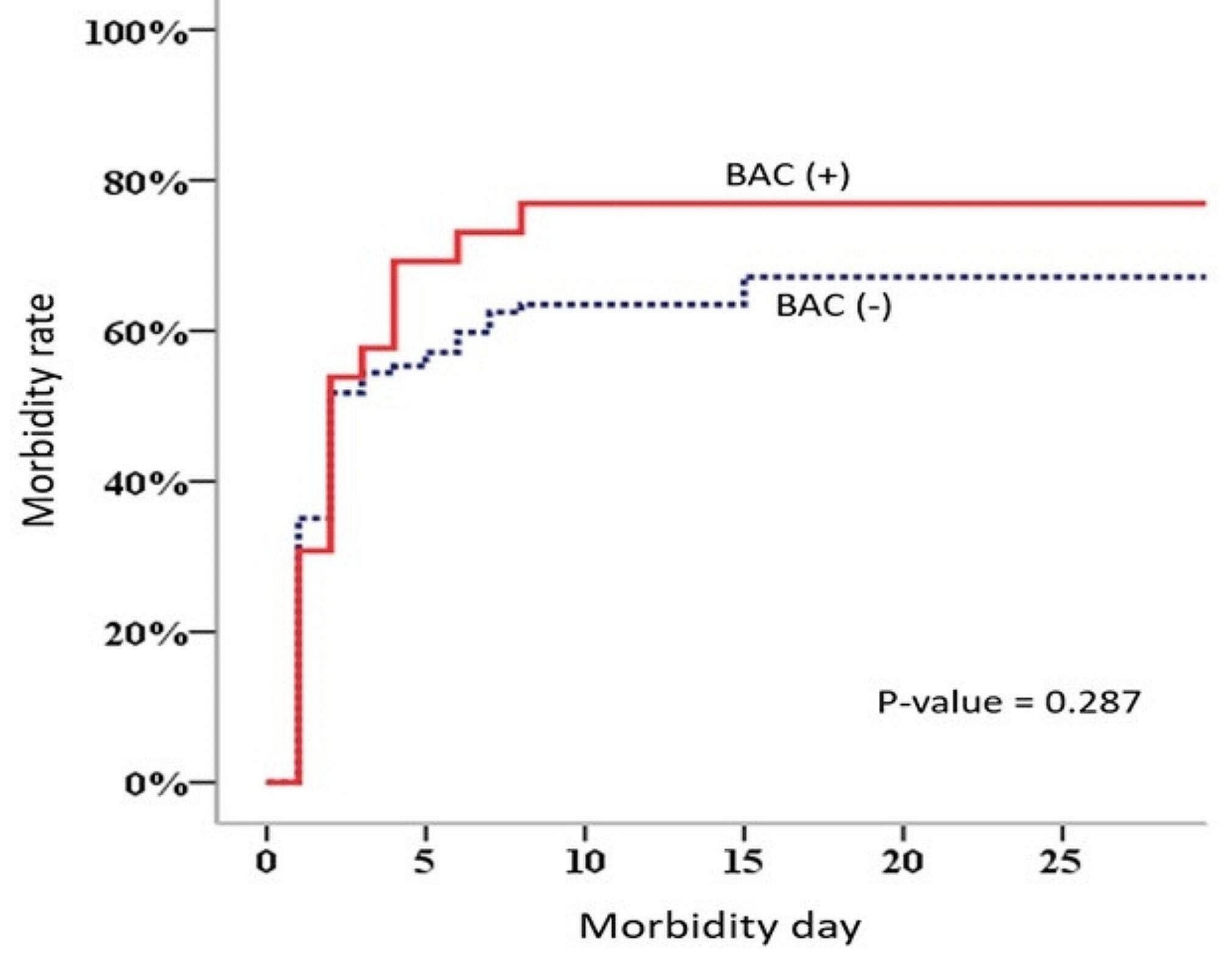
Morbidity rate trend among patients who encountered surgical blunt bowel mesentery injury in BAC-positive and BAC-negative groups BAC: blood alcohol concentration

The development of post-injury complications was not significantly different between the groups, except for unplanned intubation on admission and wound dehiscence, which were significantly more prevalent in the BAC-positive group than in the BAC-negative group (23.1% vs. 6.9%, *p* = 0.023 and 19.2% vs. 2.6%, *p* = 0.005, respectively). According to the Kaplan–Meier analysis results, patients in the BAC-positive group sustained complications without significant differences compared to those in the BAC-negative group (*p =* 0.287) (Fig. 3).

## Discussion

Despite the frequent combined presence of alcohol and trauma, previous studies have not analyzed the potential effects of alcohol-related peritonitis in a clinical setting. To the best of our knowledge, our study is the first human study to analyze the influence of AAI on patients with hemorrhage and peritonitis in a selected BBMI cohort. Benson et al. [19] screened 28,354 trauma cases with blunt and penetrating injury mechanisms. They had been tested for alcohol and required emergency laparotomy within 24 h of presentation, and their study showed that patients who were BAC-positive had a higher risk of requiring ICU care and ventilator after controlling for all confounders. The authors also reported no differences in morbidity and mortality between the BAC-positive and BAC-negative groups, which is similar to the findings of our study. However, they did not mention the baseline physiologic and hemodynamic status, clinical presentation, and associated injury of the patients in the study; therefore, they could not demonstrate the acute influence of alcohol on the clinical presentation that trauma surgeons might frequently encounter in patients requiring emergency laparotomy.

Our study cohort had a predominance of men in the BAC-positive group, which is consistent with that of previous studies in Taiwan [2, 6, 25] and in most other countries [7–11, 19, 27], indicating that alcohol intoxication is more common among men than in women with trauma. Another reason for the difference in sex distribution may be explained by the fact that drunken car driving in southern Taiwan is more common among men [25], which also reflects the higher incidence of car accidents in the BAC-positive group in our study. Although sex-and injury mechanism-specific differences were observed in the present study, we only focused on the setting of BBMI, which may have helped in decreasing the heterogeneity.

Our study indicates statistically significant lower SBP, worse SI, lower rates of WBCs ≥ 12,000 cells/mm^3^ and WBCs ≥ 12,000 or ≤ 4,000 cells/mm^3^ initially at the ED and a low incidence of transfused or packed RBCs at the ED in the BAC-positive group, suggesting that AAI not only impaired early hemodynamics but also impaired immunomodulation in patients with trauma with BBMI at the ED. The significantly lower SBP in the ED in the BAC-positive group was consistent with the findings of most clinical studies [2, 7, 10, 12]; therefore, it may be plausible to find that the SI was significantly higher in the BAC-positive group in our study because it is defined as the ratio of HR to SBP. The causes of lower SBP in alcohol-intoxicated patients are complex [13, 21] and may include a blunted noradrenergic effect, dampened neuroendocrine activation, and limited physiological reserve in response to blood loss or peritonitis. In addition to the effect of alcohol on hemorrhagic shock, Hosokawa et al. [22] used an ovine sepsis model of fecal peritonitis and concluded that AAI resulted in an earlier occurrence of hypotension, lactic acidosis, and renal insufficiency. With regard to SI, this has been studied extensively in trauma studies for the purpose of early recognition of shock or predicting the need for transfusions or likely mortality [23]. A systemic review [28] concluded that SI > 0.9 had been the most commonly accepted value for predicting post-traumatic critical bleeding, but another study suggested that SI ≥ 0.83 was the best cutoff value for predicting trauma severity measures. Besides, some authors [29] used SI combined with SIRS criteria to predict early sepsis in the ED and reported that SI ≥ 0.7 had a 3-fold increased odds of hyperlactatemia when compared to patients with SI < 0.7, whereas SI ≥ 1.0 was the most useful predictor for 28-day mortality and hyperlactatemia. Despite the presence of the evidence associated with SI and critical illness, the relationship between SI, alcohol effect, and trauma is unclear. Gustafson et al. [30] indicated that the existence of ethanol would influence the clinical presentation, outcomes, and SI with the elevation of lactate or base deficit in trauma admissions, whereas Afshar et al. [31] demonstrated that the clinical presentation and outcomes of patients with trauma could not be analyzed without considering the different BAC, and indicated that patients with undetectable BAC were associated with the significantly lowest proportion of SI ≧ 1. Moreover, a previous study which included 2490 adult trauma transfused patients [23], when using SI to predict massive transfusions within 24 h of arrival at the ED, demonstrated that SI would still have significant discriminating power in patients regardless of alcohol intoxication. This supports our findings that the BAC-positive group experienced more critical physiological dynamics than the BAC-negative group.

It is generally reported that alcohol intoxication can induce an immunosuppressive status following traumatic injury, as corroborated not only by in vivo or in vitro studies but also by clinical evidence [16, 17, 32]. In animal models, alcohol intoxication has been associated with a marked increase in lung and spleen expression of tumor necrosis factor, leading to the attenuation of circulating neutrophil function after hemorrhagic shock, which is also conducive to secondary infection challenges, either systemic or local [21]. Clinically controlled studies indirectly support this hypothesis. Gentilello et al. [15], in a study of 365 patients with penetrating abdominal trauma, found that acute alcohol intoxication (> 200 mg/dL) had a transient immunosuppressive effect with an increase in trauma-related infections. Plurad et al. [9], in an analysis of 3025 injured drivers, reported an association between the presence of alcohol (≥ 0.08 g/dL) in severely injured patients (ISS > 15) and higher incidence of sepsis, despite significantly better adjusted survival rates. In addition to the above-mentioned indirect evidence, some direct evidence from clinical studies has also postulated the theory of immunosuppression. Wagner et al. [16] demonstrated that AAI is associated with lower systemic interleukin-6 levels and leukocyte counts in patients with severe TBI in the ED. Relja et al. [17] also reported that patients with major trauma (ISS > 16) and alcohol consumption (> 50 mg/dL) had lower systemic IL-6 levels and reduced leukocyte numbers upon arrival at the ED. Even in non-trauma-critical patients, regardless of infection at admission, AAI remained significantly related to lower levels of chronic reactive protein (CRP) and WBC [32]. Despite the above-described presentation of alcohol-intoxicated patients developing post-illness reduced leukocyte counts, our data did not support this finding. However, although not statistically significant, there was a trend towards a lower number of leukocytes in the BAC-positive group. Another reason for not reaching statistical significance may be due to the BAC-negative group having an extra three patients with WBC ≤ 4000 cells/mm^3^, which may result from the responsiveness to SIRS, in contributing to the underestimate of the original mean WBC. This may explain the missing significant association between AAI and decreased leukocytes in the present study, which suggests that AAI obscures infection in the BAC-positive group because, even after alcohol peritonitis, WBC values in the BAC-positive group did not increase significantly. On the other hand, it is interesting to find that our result had a significantly lower percentage of WBC ≥ 12,000 cells/mm^3^ and WBC ≥ 12,000 or WBC ≤ 4,000 cells/mm^3^ in the univariate analysis in the BAC-positive group and remained significant after controlling for confounders, which reflected the immune-suppressive effects of alcohol and the anti-SIRS feature on arrival at the ED. In theory, the leukocyte count should increase in response to the peritonitis resulting from either alcohol or bowel injury. Similar to our data, Relja et al. [17] examined the anti-inflammatory property of alcohol in major trauma patients and found that there was a trend toward a lower incidence of SIRS (14.3% vs. 20.0%) in the BAC-positive group who were in the ED temporarily. Contrary to the data concerning the effect of alcohol on SIRS, Zeckey et al. did not support this theory in patients with multiple trauma [5]. In summary, to the best of our knowledge, this is the first study to examine the effects of positive alcohol levels on leukocytes of patients with peritonitis due to BBMI compared to patients with BBMI who were not intoxicated with alcohol.

Another interesting finding deserving mention in our study is that the BAC-positive group had significantly lower need for, and amount of packed RBC transfused at the ED, which is contrary to the negative impression of the effects of alcohol on intoxicated BAT victims. A previous investigation was reported by Rappaport et al. [33], who found that isolated blunt splenic injury patients with AAI had significant abnormal clotting and more blood transfusion requirements in the first 24 h. However, this finding was inconsistent with other recent studies, which documented a trend towards or significantly reduced consumption of blood products within the first 24 h for the BAC-positive group compared with the BAC-negative group [16, 17, 27, 34]. Our study demonstrated significantly lower rates and requirements of packed RBC at the time of ED, whereas these became insignificant subsequently in the first 24 h in the BAC-positive group, suggesting that AAI may impair the acute stage of trauma victims regarding the possible mechanisms of attenuation of coagulopathy or regulation of hemodynamics.

With regard to the mitigation of coagulopathy, Lustenberger et al. [8] reported that alcohol was an independent protective factor for early admission coagulopathy in AAI patients with severe TBI, contributing to a neuroprotective effect with significantly lower incidence of mortality in BAC-positive patients compared to their BAC-negative counterparts. This is also supported by Howard et al. [27], who reported that alcohol was not only a negative predictor of coagulopathy by a traditional INR > 1.3, but was also not correlated with 24 h-transfusion in patients with trauma. They thought that alcohol may have a bidirectional effect on coagulation (impaired clot formation and decreased fibrinolysis in rotational thrombo-elastometry) [34] and found that patients with abnormal thromboelastography at admission, and BAC-positive patients had significantly lower transfusion requirements and rates within the first 24 h than those in their BAC-negative counterparts. This could be explained by the hypercoagulable effect of alcohol in our data and why the BAC-positive group had significantly lower rates and blood requirements at the ED in the absence of differences in the incidence of post-injury coagulopathy between the two groups, using the traditional standard INR measures. Another possible reason for this observation may be related to the hemodynamics. Animal studies have provided supporting evidence that ethanol would be more sensitive to hemorrhage [21]. Molina et al. conducted rat experiments with both fixed pressure (40 mmHg) and fixed volume (50%) hemorrhagic shock models and concluded that alcohol decreased the amount of blood loss necessary to reach or maintain the shock state, whereas alcohol elicited a greater level of hypotension early after removing half of the blood volume. Similarly, alcohol intoxication leads to the early development of shock in an ovine fecal peritonitis model [22]. Accordingly, we thought that patients with AAI would exhibit shock early in the ED, thereby attracting our attention, allowing them to receive earlier resuscitation or fewer blood products to restore hemodynamic stability. On the contrary, BAC-negative patients often suffered from subtle unclear bleeding and subsequently needed more resuscitation or blood products once the shock occurred. Overall, although the cause is unclear, one hypothesis suggests that alcohol may play a critical role in blood transfusion during the early resuscitation period because it decreases the need for transfusion in the ED. Future clinical studies underlining the potential effects of alcohol on pathophysiological mechanisms are needed to confirm our results.

Based on the aforementioned studies, the impact of acute alcohol intoxication (AAI) on outcomes in traumatic patients, particularly those with alcoholic peritonitis, remains unclear. The discrepancies in findings may be attributed to variations in methods, diverse study populations with different cut-off blood alcohol concentration (BAC) levels, and varying study management conditions. In our previous retrospective study [6], which investigated the effect of AAI on adult trauma admissions, it was concluded that although patients with positive BAC had a significantly higher mortality rate than those with negative BAC, AAI did not significantly influence mortality after employing propensity score-matching methods to account for sex, age, comorbidity, and injury severity. This suggests that higher mortality is likely associated with patient characteristics or injury severity. This perspective is supported by Benson et al. [19], who, through logistic regression analysis, found no significant correlation between alcohol and mortality or hospital length of stay in trauma patients requiring emergency laparotomy, after controlling for age, sex, Injury Severity Score (ISS), Glasgow Coma Scale (GCS), and injury mechanism. In contrast, another of our previous studies [25] focusing on alcohol-related admissions in trauma patients revealed that patients with positive BAC had lower ISS and NISS and shorter hospital stays. Additionally, our institution’s analysis [24] of car driver accidents associated with AAI effects on driving under the influence, following a reduction in the legal BAC limit from 50 to 30 mg/dL in Taiwan, showed that driving under alcohol influence increased the odds ratios of mortality from 5.6 to 4.3 times after the legal BAC reduction, failing to significantly decrease mortality in drivers. Some authors [31] further addressed the implication that the effect of AAI on the odds of mortality and injury severity exhibited a U-shaped relationship rather than a tangential one. Accordingly, the AAI effect on the outcomes of trauma patients seems to be complex, further prospective studies assessed alcoholic peritonitis after trauma are still needed.

The outcomes of our current study are in agreement with those of other investigators [5–7, 12, 16, 17, 19, 27, 34], indicating that BAC-positive patients experience similar injury patterns with the majority of morbidities and mortality, compared to the BAC-negative group. Regarding morbidity, BAC-positive patients appeared to have higher rates of unplanned intubation in the ward and wound dehiscence than BAC-negative patients. The study by Benson et al. [19] supported our findings in that they reported that in BAC-positive patients requiring emergency laparotomy, unplanned intubation was the only complication with increased odds (1.38 times), after they had performed a regression analysis that included all postoperative complications. Further analysis of mortality in our data showed that although the BAC-positive group had similar overall mortality, they had a higher incidence of bowel-related mortality than the BAC-negative group. Accordingly, we thought that the above-mentioned significant differences in morbidity and mortality may be due to malnutrition where the BAC positive group lacked the capacity to provide enough immunomodulation during the critical period of recovery and tissue repair, as a result of chronic alcoholism. This immunodeficiency could be due to liver disease or inadequate intake of vitamins and/or essential minerals. Although our study did not provide data regarding the history of chronic alcohol consumption, chronic alcohol abuse is likely to occur at a higher incidence in injured victims with AAI. This finding was supported by the evidence of Rivara et al. [35] who included 2,657 trauma admissions with 47% alcohol intoxication and found that 75% of those intoxicated were chronic alcohol abusers. Finally, the results of the current study indicate that although AAI initially influenced hemodynamic parameters, the effect may be transient. While facing AAI victims, besides paying more attention to patients in the acute resuscitation stage, we cannot neglect subsequent postoperative care, especially nutrition support.

This study has some limitations that should be considered when interpreting the results. First, this was a single-center retrospective study with a small sample size, which limits the completeness of the data and statistical power. Second, our data did not contain information on comorbidities, alcohol consumption duration, or history of alcohol dependence, which could affect the results of this study. Patients with alcohol abuse have higher in-hospital morbidity and mortality [14]. Third, no routine drug screening was performed; hence, the possibility of an association with other drugs could not be excluded. Fourth, the study did not include information on the patients who died at the scene, which may have biased the results. Patients who engage in drunk driving usually have lower compliance with safety device use and very high BAL incidence [2, 11, 24]. Finally, although alcohol consumption can be evaluated as a continuous variable, we analyzed it as a categorical variable. There is evidence from a prior study [31] where Afshar et al. reported that different BAC groups had different injury severities and mortalities, and noted that the association between BAC and clinical outcomes cannot be analyzed without considering the different alcohol levels. Despite these limitations, the present study is the first human study to investigate the influence of AAI on patients with concomitant hemorrhage and peritonitis in terms of the simultaneous evaluation of SI, leukocytes, and blood transfusions, and could provide further insights into the impact of alcohol on initial resuscitation in patients with BAT.

## Conclusions

This study included data on patients with BBMIs collected over a period of 13 years and analyzed the impact of alcohol consumption on peritonitis and outcomes in patients with BBMI. Our results indicate that patients with BBMI and acute alcohol consumption have lower blood pressure, higher shock index, and disrupted inflammatory status at the time of emergency department presentation. Furthermore, the bowel-related mortality rate is higher. In the light of these findings, routine alcohol testing is recommended in the ED, and more attention should be paid to guiding the early resuscitation of patients with BAT and alcohol consumption.

## Data Availability

Data were obtained from Chang Gung Research Database and are available by corresponding with the author and obtaining permission.

## Abbreviations

AAI: acute alcohol intoxication
AIS: abbreviated injury score
BAC: blood alcohol concentration
BAT: blunt abdomen trauma
BBMI: blunt bowel mesentery injury
BT: blood transfusion
ED: emergency department
HR: heart rate
ICU: intensive care unit
INR: international normalized ratio
ISS: injury severity score
SIRS: systemic inflammation response syndrome
TBI: traumatic brain injury
WBC: white blood cell
